# Information Theory in Molecular Evolution: From Models to Structures and Dynamics

**DOI:** 10.3390/e23040482

**Published:** 2021-04-19

**Authors:** Faruck Morcos

**Affiliations:** 1 Department of Biological Sciences, University of Texas at Dallas, Richardson, TX 75080, USA; faruckm@utdallas.edu; 2 Department of Bioengineering, University of Texas at Dallas, Richardson, TX 75080, USA; 3 Center for Systems Biology, University of Texas at Dallas, Richardson, TX 75080, USA

Historically, information theory has been closely interconnected with evolutionary theory. The work of Ronald Fisher in population genetics [1] and the formulation of the principle of minimum Fisher information [2] are just two early examples of such connections. In recent years, with the advent of high-throughput sequencing technologies, the field of molecular evolution has been able to take advantage of large amounts of samples from evolution to improve models and applications to understand structural, dynamical, and functional aspects of biomolecules. Information metrics have been prevalent, in recent years, to estimate the likelihood that two amino acid sites in a protein are coevolving. A relevant example of such metrics is *Direct Information* (DI) [3,4] used in the context of Direct Coupling Analysis to estimate if two positions in a multiple sequence alignment are likely to be proximal in the 3D structure of a protein or RNA molecule. Other standard information metrics like *Mutual Information* have been applied and are particularly useful for the case of molecular complexes and interactions [5,6].

This special issue focuses on important aspects of the study of molecular evolution through the statistical features of sequence data, molecular simulation, and evolutionary convergence towards specificity in signaling networks. Three articles [7,8,9] investigate how phylogenetic relationships in sequence data have an effect in the inference procedure of a joint probability distribution $P(a_{1},a_{2},a_{3},\ldots ,a_{L})$ of a given sequence of length *L*. Particularly, these studies are centered under the premise that a preprocessing step for multiple sequence alignment analysis might reduce phylogenetic bias and could improve the inference procedure. These methods, ultimately, improve prediction of amino acid contacts and functional connections among amino acid sites.

In [7], Hockenberry et al. conducted a systematic study of previously relevant reweighting schemes that have been useful in other applications. These methods contrast versus the current practices of identity-based sequence reweighting used in Potts model inference. They find that previous applications do not add considerable value for the inference task and leave open the question for novel schemes that might improve the inference of coevolving residue pairs. Interestingly, in [8,9], the authors propose novel schemes to account for phylogenetic bias. First, Horta et al. [8] introduce a new inference method which uses a priori information about phylogeny to enhance contact prediction and fitness effects in simulated data. Second, Maliverni et al. [9] propose another scheme called continuous sequence reweighting (SR) that reveals structural features that are unique to subfamilies as opposed to determining global properties common to all family members. These articles as a whole provide an in-depth and useful picture on how to deal with phylogenetic correlations in the task of contact inference and the estimation of the effects of mutation.

A second set of articles in this issue [10,11,12] deals with the complex problem of evolutionary dynamics in protein structures and sequences. Cadet et al. [12] study formal statistical properties of sequence change and show how fluctuations follow a −5/3 Kolmogorov power and behave like an incremental Brownian process. In another study, Wang et al. [10] investigate members of the family of β-Lactamases, enzymes involved in antibiotic resistance. In this study, they uncovered, via molecular simulations, important amino acid positions that share functional and dynamical features with another class of evolutionarily related proteins called Penicillin-binding proteins (PBP), enhancing our understanding of the dynamics of catalytic residues in the context of antibiotic resistance. In a third article, also concerned with the dynamics of protein evolution, Campitelli et al. [11] devise accurate metrics to quantify epistasis upon amino acid perturbations (EpiScore) and the asymmetric Dynamic Coupling Index (DCIasym) to measure how connected residues are affected depending on which residue has been perturbed. These metrics are relevant contributions to the study of allostery and the evolutionary forces that shape this important functional phenomenon.

In a final study, Sinner et al. [13] construct another information metric to predict the degree of specificity between molecules in two-component signaling networks. Molecular interactions between histidine kinases (HK) and response regulators (RR) have evolved towards amino acid specificity at the physical interface in the HK-RR complex where phosphotransfer occurs. A degree of coevolutionary strength at this interface can be quantified for a large number of organisms. The authors created a public web server called ELIHKSIR.org (Evolutionary Links Inferred for Histidine Kinase Sensors Interacting with Response regulators) to facilitate the prediction and analysis of these links and to assess the effect of mutations in interacting specificity.

All together, the methodological contributions presented in this issue of *Entropy* will help advance the study of molecular evolutionary dynamics through the lens of information theoretical metrics and a combination of structural modeling and molecular dynamics simulations.
